# Clinical Implications of the Coexistence of Anemia and Diabetes Mellitus in the Elderly Population

**DOI:** 10.1155/2021/8745968

**Published:** 2021-10-18

**Authors:** S. S. Michalak, E. Wolny-Rokicka, E. Nowakowska, M. Michalak, L. Gil

**Affiliations:** ^1^Department of Pharmacology and Toxicology, Collegium Medicum, University of Zielona Gora, Zielona Gora, Poland; ^2^Department of Radiotherapy, Multidisciplinary Hospital, Gorzow Wielkopolski, Poland; ^3^Department of Computer Science and Statistics, Poznan University of Medical Sciences, Poznan, Poland; ^4^Department of Hematology and Bone Marrow Transplantation, Poznan University of Medical Sciences, Poznan, Poland

## Abstract

Diabetes mellitus (DM) and also anemia are common in the elderly and have a negative impact on the clinical outcomes of patients. The coexistence of anemia and DM seems to be insufficiently recognized; therefore, the aim of our study is to analyze the incidence and clinical consequences of this coexistence, including mortality, in the population of people aged ≥60. A retrospective study was conducted on 981 primary care clinic patients aged ≥60 during 2013-2014. The prevalence of coexistence of DM and anemia (defined in accordance with WHO) and data on the incidence of comorbidities, hospitalization, medical procedures, and all-cause mortality were analyzed. In the study population, 25% had DM, while 5.4% had both DM and anemia. Peripheral artery disease (PAD) was found in 48 patients (4.89%) of the entire study population, more often in men (*p* < 0.001). Diabetic patients with anemia compared to nonanemic diabetics had more comorbidities (median 4 (4, 5) vs. 3 (2–4); *p* < 0.001)—PAD more often (*p* = 0.004), more hospitalization (median 2 (0–11) vs. 0 (0–11); *p* < 0.001), and more frequent medical procedures (e.g., percutaneous coronary intervention (*p* < 0.001), coronary artery bypass surgery (*p* = 0.027), arteriography (*p* < 0.001), and bypass surgery or endovascular treatments of lower limb ischemia (*p* < 0.001)). The cumulative survival of patients with both DM and anemia vs. nonanemic diabetics at 36 months was 86.4% vs. 99.3% (*p* < 0.001). A multivariate logistic regression model showed anemia to be a significant risk factor for death in diabetic patients (*p* = 0.013). Patients with both DM and anemia have more comorbidities than nonanemic diabetic patients; they are more often hospitalized, require medical procedures more frequently, and are at a higher risk of death. Effective treatment of anemia in patients with DM is advisable and may well improve the prognosis of patients.

## 1. Introduction

Both diabetes mellitus (DM) and anemia are common in the elderly population, and both are associated with the severity of the course of many comorbidities (especially cardiovascular diseases) and an increase in the risk of death [1–5]. In 2019, 463 million people worldwide were diagnosed with diabetes and approximately 20% of them were in their 60s [6]. The prevalence of anemia in the elderly population ranges from about 3% to over 60% [7]. The long-term course of diabetes leads to micro- and macrovascular damage, which negatively affects the circulatory system, nervous system, kidneys, and eyes [8]. DM is a known factor influencing the development of atherosclerosis and as a result the course of diseases such as peripheral arterial disease (PAD) or coronary heart disease. Anemia also, especially in elderly patients, has a negative impact on the circulatory system and leads to left ventricular hypertrophy [9], exacerbation of symptoms of ischemic heart disease, heart failure [10, 11], or the formation of atherosclerotic changes in the vessels [12]. The most common etiologies of anemia in the elderly are anemia of chronic diseases (ACD), unexplained anemia, and iron deficiency anemia [13]. A significant group of elderly anemic patients remains untreated due to the lack of a known cause of the decrease in hemoglobin (Hb) [14]; hence, anemia can negatively affect health for many years.

The coexistence of anemia and diabetes is not often discussed in literature, and this problem seems to be insufficiently recognized [15]. Therefore, the aim of our study is to analyze the frequency of the simultaneous occurrence of anemia and DM and to study the clinical consequences of this coexistence, including mortality in the population of people aged ≥60.

## 2. Materials and Methods

This is a retrospective analysis of the data of patients aged ≥60 years who were under the care of the primary care clinic in Poland for the years 2013-2014. The collected data (laboratory tests, diseases, treatments, hospitalizations, and medical procedures) come from the medical records of primary care clinics, specialist consultation cards, and patient discharge records. The patients' data after the age of 60 were analyzed.

Anemia was defined according to WHO criteria (Hb < 13 g/dL in men, Hb < 12 g/dL in women) [16]. The definitions of the types of anemia, severity, and definitions of diseases were presented earlier [1]. Diseases were defined in accordance with the recommendations of international societies which were in force at the time of diagnosis [17–27]. DM was diagnosed on the basis of random blood glucose levels ≥ 11.1 mmol/L (200 mg/dL), in the presence of typical symptoms of diabetes (such as increased thirst, polyuria, and weakness) or twice confirmed blood glucose after fasting ≥ 7.0 mmol/L (126 mg/dL), blood glucose values ≥ 11.1 mmol/L (200 mg/dL) at 120 minutes after an oral glucose load of 75 g. Using antidiabetic drugs and/or insulin confirmed DM [28]. DM with complications was defined as diabetes with the presence of at least one of the macroangiopathic complications (presence of ischemic heart disease, lower extremity ischemia, and stroke history) or microangiopathic complications (presence of diabetic nephropathy, diabetic neuropathy, diabetic retinopathy, and diabetic foot). Complicated arterial hypertension was diagnosed in the presence of subclinical or clinical signs of organ damage in the course of arterial hypertension.

The frequency of the following medical procedures was recorded: coronary angiography, percutaneous coronary intervention, coronary artery bypass grafting, and device therapy (implantable cardioverter defibrillator (ICD), cardiac resynchronization therapy (CRT)). The frequency of lower extremity amputations due to ischemia and other invasive treatments of lower extremity arterial disease were also analyzed: bypass surgery and endovascular treatment (percutaneous angioplasty with stenting, balloon angioplasty).

Data on all-cause mortality in patients between January 2013 and December 2014 were obtained from reports by the National Health Fund. In death, analysis of the risk factors in diabetic patients, various details were taken into account: age, presence of anemia, selected comorbidities, some laboratory tests, history of acute coronary syndrome before age 60, and stroke before age 60.

The study was compiled in accordance with the Declaration of Helsinki and was approved by the Bioethical Committee of Poznan University of Medical Sciences.

### 2.1. Statistical Analysis

Interval data is presented as means and standard deviations. In this case, the data does not follow normal distribution; the descriptive statistics are presented as medians and interquartile ranges. The numerical data was compared using the Mann–Whitney test. Categorical data were analyzed by the chi-square test, and its results are presented as numbers and percentages or odds ratios (OR) and 95% CI. To find significant factors that may increase the risk of occurrence of anemia in diabetic patients, a logistic regression was performed. The results are presented as OR and 95% CI. The logistic regression was performed both as a univariate and multivariate model. The survival was performed with the Kaplan-Meier estimate. The differences between survival curves were analyzed by the log-rank test. The influence of the studied parameters on the risk of death was done by the Cox proportional hazard model. The results are presented as hazard ratio (HR) and its 95% CI.

The analysis was performed using the statistical package TIBCO Software Inc. (2017); Statistica (data analysis software system), version 13 (http://statistica.io). All tests were considered significant at *p* < 0.05.

## 3. Results

**Figure 1:**
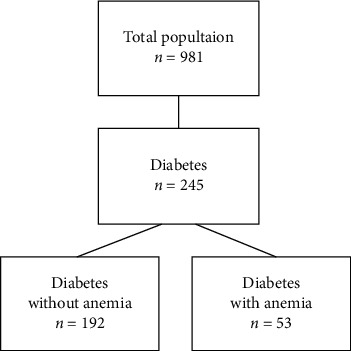
Study population.

In the group of 981 patients in the study (aged ≥60 years), there were 245 diabetic patients (140 women; 105 men)—24.97% of the entire study population (Figure 1). The majority of the diabetic group had type 2 DM (81%), of whom 47% were receiving insulin.

DM with complications was found in 143 patients (74 women: 51.75%; 69 men: 48.25%)—58.37% of the diabetic group. 53 patients had both anemia and DM, including 40 (75.47%) patients with DM with complications, whereas the diabetics without anemia amounted to 192 patients (of whom 103 (53.65%) have DM with complications). The demographic characteristics of the patients with DM are presented in Table 1.

The patients with DM with complications were more than 2.5 times more likely to have anemia (OR = 2.659; 95% CI 1.337–5.286; *p* = 0.004). Women with DM with complications suffered from anemia 4 times more often than men (OR = 4.231; 95% CI 1.594–11.23; *p* = 0.002).

### 3.1. Comorbidities

The group with both DM and anemia had more comorbidities and was more often hospitalized compared to the nonanemic diabetic patients (respectively; *p* < 0.001) (Table 2).

The clinical characteristics of diabetic patients are presented in Table 3. Significant differences were found only in the case of PAD (*p* = 0.004) and hypertension with complications (90.6% of patients with both DM and anemia; 63.5% of patients with DM without anemia) (*p* < 0.001). In the entire study population of 981 patients, PAD was found in 48 (4.89%) patients, including 17 (2.86%) women and 31 (8.01%) men (*p* < 0.001). In the group of patients with DM, PAD was found in 18 (17.14%) men and 7 (5%) women (*p* = 0.002).

### 3.2. Medical Procedures

In the anemic diabetic group, the following procedures were conducted significantly more often: coronary angiography (*p* < 0.001), percutaneous coronary intervention (*p* < 0.001), coronary artery bypass surgery (*p* = 0.027), arteriography (*p* < 0.001), and invasive treatment of lower extremity ischemia (bypass surgery or endovascular treatments; *p* < 0.001) (Table 4). Arteriography and coronary angiography were performed significantly more often in diabetic patients in comparison with nondiabetic patients (*p* = 0.027).

### 3.3. Characteristics of Anemia in the DM Group

The three most common types of anemia in the diabetic group were unexplained anemia (32.1%), anemia of chronic diseases (28.3%), and iron deficiency anemia (18.9%). Other types of anemia were presented as follows: chemo- and/or radiotherapy-induced anemia (9.4%), renal insufficiency anemia (3.8%), vitamin B12 deficiency anemia (1.9%), iron and vit. B12 deficiency anemia (1.9%), vit. B12 and folate deficiency anemia (1.9%), and hemorrhagic anemia (1.9%).

Mild anemia was the most prevalent (71.7%); the others were moderate anemia in 22.6%, severe in 3.8%, and very severe in 1.9%.

### 3.4. All-Cause Mortality

Of the 245 diabetic patients, 7 (2.86%) died during the 36-month follow-up, including 6 (11.32%) in the anemic diabetic group and 1 (0.52%) in the nonanemic diabetic group.

**Figure 2:**
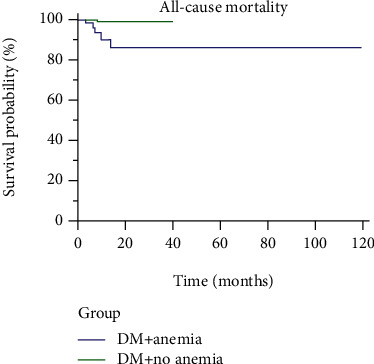
Cumulative survival probability of the anemic and nonanemic diabetic patients.

Cumulative survival probability rate after 36 months in both groups, anemic diabetics vs. nonanemic diabetics, was 86.4% vs. 99.3%, respectively (*p* < 0.001) (Figure 2).

### 3.5. Risk Factors for Death in a Diabetic Patient

A number of risk factors for death in diabetic patients were examined (Table 5), and the multivariate regression analysis showed anemia to be the only significant risk factor (*p* = 0.013).

## 4. Discussion

The prevalence of diabetes is increasing worldwide, and the problem is repeatedly referred to as the diabetes pandemic [29]. The estimated global average prevalence of DM in elderly patients was 19.3% in 2019 [30]. In our study population of 981 outpatients aged ≥60, one quarter of patients suffered from DM and the coexistence of anemia and DM was found in 21.6% of patients. This is in accordance with the studies conducted in China (22%) [31], Australia (23.3%) [32], and Ethiopia (19%) [33]. Some studies reported more frequent simultaneous occurrence of anemia and DM, e.g., hospitalized patients in Ethiopia (34.8%) [34] and endocrinology department outpatients in Iran (30.4%) [35]. Even higher incidence was recorded in Tobago (in the Caribbean) (46.5%) [36], significantly higher in the group of outpatients aged ≥75 in the United Kingdom (59%) [37], and the highest in hospitalized patients in the internal medicine department in Pakistan (63%) [38].

Differences in the frequency coexistence of anemia and DM may be due to a number of factors. Based on the current literature, it can be concluded that this coexistence is more often found in patients who are hospitalized or patients under the care of specialized clinics. The age of the studied patients is of high importance (the prevalence of anemia and DM increases with age) as well as their race (the Negroid race is more prone to the coexistence of both diseases). Other possible factors are the duration of anemia, the type of DM, and coexistence and severity of CKD. The level of health care, the economic situation of the country in which the study was conducted, and the geographical location (altitude) are also of importance.

The pathogenesis of anemia in diabetes is multifactorial and is associated mainly with the presence of chronic inflammation, diabetic nephropathy, hyporeninemia, and nutritional deficiencies [15, 39]. Vitamin B12 and folate deficiency is frequently observed among diabetic patients [15, 40]. Erythropoietin (EPO) deficiency and/or resistance, iron deficiencies (resulting from reduced dietary intake, impaired enteral absorption, blood loss), and proteinuria (with loss of transferrin or EPO) are mechanisms leading to anemia development in patients with diabetic kidney disease [41, 42]. In men with type 2 DM, obesity, and insulin resistance, low testosterone levels are often found, which may also increase the risk of anemia [43]. In the course of DM, especially type 2, the expression of proinflammatory cytokines (mainly IL-6) and CRP increases [44]. Both anemia and DM are associated with inflammation, and our study showed that patients with anemia and DM more frequently presented increased ESR. Hyperglycemia and obesity are important contributors to inflammation, which result in EPO deficiency or lack of iron availability for erythropoiesis. Apart from exacerbating inflammation, hyperglycemia can also affect cardiovascular complications, e.g., increase the size of some types of infarct [45]. The common coexisting conditions in type 1 diabetes are autoimmune diseases (especially celiac disease, Addison's disease, Hashimoto's thyroiditis, and autoimmune gastritis) which may lead to anemia [15, 46]. Diabetic acidosis promotes the occurrence of hemolytic anemia in patients with congenital deficiency of glucose-6-phosphate dehydrogenase or in sickle cell anemia [47]. The treatment of diabetes can also contribute to the onset of anemia, e.g., the use of biguanides [48], thiazolidinediones [49], and renin-angiotensin aldosterone (RAA) system blockers [50]. Data from a randomized controlled trial determined the association between the use of metformin and the risk of anemia in type 2 DM. The mechanism of this phenomenon remains unknown so far, and vitamin B12 deficiency does not seem to be the only reason [51]. However, not all diabetic patients are anemic. Certain correlation between insulin resistance and erythropoiesis has been observed. Patients with a higher insulin resistance had a higher erythrocyte count [52]. A proper diet and treatment of DM can reduce inflammation and thus have a positive impact on erythropoiesis and atherosclerotic progression. Metformin combined with a low-calorie diet has been shown to reduce the inflammation and oxidative stress associated with obesity and insulin resistance in prediabetic patients [53]. GLP-1 therapy reduces the inflammation and oxidative stress of atherosclerotic plaque, which probably affects its stability [54]. Sodium glucose cotransporter-2 (SGLT2) inhibitors can improve erythropoiesis, reducing the risk of anemia or the need to use erythropoiesis-stimulating agents (ESA) [55]. Canagliflozin in diabetic patients with nephropathy leads to an increase in reticulocyte count, erythrocyte count, and hematocrit [56], which is mediated by EPO [56]. SGLT2 inhibitors may also counterbalance the negative effects of RAA system blockers on erythropoiesis [50].

Anemia leads to hypoxia and the impairment of many tissues and organs, especially the circulatory and renal. Poorer cardiovascular and renal functions, in turn, are factors contributing to anemia [41, 42, 57]. This results in a “vicious cycle” and seems to be an important factor in the coexistence of anemia and diabetes. Additionally, anemia causes falsely low levels of glycosylated hemoglobin in diabetic patients and as a consequence of a lack of proper treatment, which may lead to complications of DM [58].

Strong associations of the coexistence of DM and anemia with diabetic nephropathy, neuropathy, and retinopathy [35, 59] as well as a significant number of comorbidities with this coexistence [60–62] have been reported previously. The links between anemia and diabetes and the complications can be partially explained by the influence of EPO, which apart from affecting the hematopoiesis has a number of other metabolic effects. EPO receptors have been detected in various tissues, especially in adipose tissue, pancreas, brain tissue, and in the retina. It is believed that the presence of EPO receptors in the hypothalamus regulates metabolism of glucose, while in adipose tissue it has an anti-inflammatory effect and increases insulin sensitivity [63]. However, additional studies to better understand the key pathomechanisms and consequences of the coexistence of anemia and diabetes are required.

Our results demonstrated that patients with DM and anemia (compared to the nonanemic diabetics) had more comorbidities (most commonly DM with complications, hypertension with complications, and PAD), were more often hospitalized, and had a higher risk of death. A decrease in Hb in diabetic patients increases the risk of hospitalization and death, as has been shown in previous studies [11, 64, 65]. Patients with DM have an increased risk of death in general from any cause [66, 67], but mainly from cardiovascular diseases [3, 59]. The main factors are the duration of DM and the age of patients [66]. In a retrospective 10-year analysis of patients over 65 years of age, the increased risk of death in diabetic patients was approximately 70% versus without DM [68]. Anemia seems to be a risk multiplier for all-cause mortality [41]. In our analysis, patients with anemia and DM were not only hospitalized more often but also more often underwent various medical procedures, such as coronary angiography, percutaneous coronary intervention, coronary artery bypass surgery, arteriography, and invasive treatment of chronic lower limb ischemia (bypass surgery, endovascular treatments). Our results cast a new light on the consequences of anemia in diabetic patients. To our knowledge, the previous studies only covered medical procedures performed in patients with either anemia or diabetes and not in the presence of both disorders. A Polish study which analyzed patients who underwent vascular angioplasty procedures showed the anemic patients had multivessel disease in angiography, more often experienced myocardial infarction, more frequently needed coronary artery bypass graft, and had a higher risk of death during the one-year follow-up [69]. Anselmino and colleagues showed that patients with DM required percutaneous coronary intervention or coronary artery bypass grafting more often than nondiabetic patients [70].

The findings of our study revealed that 4.89% of all included elderly patients had PAD and it was diagnosed more often in men than in women. Based on the literature, it is estimated that the incidence of PAD is 3-10%, is higher in men, and increases with age [27]. Our study confirms previously established data that in diabetic patients the incidence of PAD is about 2 times more frequent coexisting than in nondiabetic patients. Our research showed that patients with DM and anemia vs. nonanemic diabetics suffered from PAD more often; they more frequently had invasive procedures related to lower limb ischemia, such as bypass surgery and endovascular treatment. Invasive procedures for PAD are performed when the patient presents clinical symptoms. Anemia may worsen these symptoms. There is a mutual correlation between anemia, DM, and medical procedures. Medical procedures may affect the development and severity of anemia. While aggravating symptoms of circulatory system, anemia may be a deciding factor when evaluating the need to perform medical procedures.

Our study has its limitations, mostly due to the retrospective character of the analysis. There was no possibility to obtain some missing information, such as the duration of DM and some laboratory data. The data comes from a healthcare clinic, where there was only a small group of patients with CKD. However, the study examined almost 1000 patients and detailed data was collected on many parameters such as patients' diseases, hospitalization, medical procedures, and death.

## 5. Conclusions

The comparison of the two groups with both DM and anemia vs. nonanemic diabetics showed the following characteristics of the former group: more comorbidities, including PAD, more frequent hospitalizations, and more various medical procedures performed. Our study confirmed that anemia has a negative impact on the survival of patients with DM. We believe anemia to be one of the underrecognized risk factors for DM. Therefore, proper treatment of anemia in patients with DM is highly recommended, as it may positively affect the course of DM and the patients' prognosis. Further studies on the coexistence of anemia and diabetes are needed.

## Figures and Tables

**Table 1:** Characteristics of the diabetic group.

	Diabetes *n* = 245	Diabetes with anemia *n* = 53	Diabetes without anemia *n* = 192	*p* value
Female, *n* (%)	140 (57.1)	28 (52.8)	112 (58.3)	0.475
Age mean ± SD	71.4 ± 8.28	73.5 ± 8.51	70.8 ± 8.14	
Age median (LQ-UQ)	69.9 (64.26-77.93)	74.6 (65.53-81.02)	68.7 (63.97-77.55)	0.294

LQ: lower quartile; UQ: upper quartile.

**Table 2:** Differences in the number of comorbidities and the frequency of hospitalization.

Variable	Diabetes with anemia		Diabetes without anemia		*p* value
	Median (min–max)	Lower-upper quartile	Median (min–max)	Lower-upper quartile	
Number of comorbidities	4 (1–9)	4–5	3 (1–8)	2–4	<0.001
Number of hospitalizations	2 (0–11)	1–4	0 (0–11)	0–1	<0.001

**Table 3:** Clinical characteristics of diabetic patients with and without anemia.

Characteristics	Diabetes *n* = 245	Diabetes with anemia *n* = 53	Diabetes without anemia *n* = 192	*p* value
*Level of hemoglobin in the blood*				
Female (g/dL), mean ± SD	12.97 ± 1.76	10.09 ± 1.33	13.69 ± 0.91	<0.001
Male (g/dL), mean ± SD	13.99 ± 1.85	11.34 ± 1.43	14.81 ± 0.98	<0.001

*Other laboratory tests*				
WBC (10^9^/L), mean ± SD	7.33 ± 1.97	7.3 ± 2.27	7.34 ± 1.89	0.312
RBC (10^12^/L), mean ± SD	4.43 ± 0.60	3.7 ± 0.54	4.63 ± 0.44	0.301
PLT (10^9^/L), mean ± SD	236.37 ± 83.69	276.77 ± 130.58	225.57 ± 61.95	0.288
ESR (mm/h), mean ± SD	24.33 ± 22.80	37.56 ± 32.75	19.96 ± 16.38	0.034
CRP (mg/L), mean ± SD	24.18 ± 56.94	46.93 ± 78.07	4.57 ± 7.27	0.288

*eGFR leveln(%)*				
eGFR < 60 mL/min/1.73 m^2^	46 (20.5)	16 (34.04)	30 (16.95)	0.010
eGFR ≥ 60 mL/min/1.73 m^2^	178 (75.5)	31 (65.96)	147 (83.05)	0.010

*Comorbiditiesn(%)*				
Hypertension	232 (94.7)	51 (96.2)	181 (94.3)	0.582
Hypertension with complications	170 (69.4)	48 (90.6)	122 (63.5)	0.001
Coronary heart disease	121 (49.4)	31 (58.5)	90 (46.9)	0.265
Heart failure	33 (13.5)	17 (32.1)	16 (8.3)	0.092
Atrial fibrillation	29 (11.8)	10 (18.9)	19 (9.9)	0.495
PAD	25 (10.2)	13 (24.53)	12 (6.25)	0.004
Venous thromboembolism	6 (2.4)	2 (3.8)	4 (2.1)	0.903
Thyroid diseases	47 (19.2)	14 (26.4)	33 (17.2)	0.469
Pulmonary disease	25 (10.2)	8 (15.1)	17 (8.9)	0.640
Asthma	13 (5.3)	3 (5.7)	10 (5.2)	0.976
COPD	8 (3.3)	3 (5.7)	5 (2.6)	0.826
Chronic kidney disease	41 (16.7)	18 (34)	23 (12)	0.090
Chronic liver diseases	15 (6.1)	10 (18.9)	5 (2.6)	0.384
Rheumatic diseases	0 (0)	0 (0)	0 (0)	—
Dementia	6 (2.4)	2 (3.8)	4 (2.1)	0.903
Cancer	29 (11.8)	13 (24.5)	16 (8.3)	0.232

*Drugsn(%)*				
Insulin	60 (24.5)	20 (37.7)	40 (20.8)	0.011
Metformin	91 (37.1)	20 (37.7)	71 (37)	0.951
ACE inhibitors	111 (45.3)	38 (71.7)	73 (38)	<0.001
Aspirin	106 (43.3)	36 (67.9)	70 (36.5)	<0.001
DOACs+VKA	27 (11)	13 (24.5)	14 (7.3)	0.001

ALT: aminotransferase alanine; AST: aminotransferase aspartate; COPD: chronic obstructive pulmonary disease; CRP: C-reactive protein; DOACs: direct oral anticoagulants; ESR: erythrocyte sedimentation rate; PAD: peripheral artery disease; PLT: platelets; VKA: vitamin K antagonists; WBC: white blood cells; RBC: red blood cells.

**Table 4:** Comparison of the frequency of selected medical procedures in different patient groups.

Procedures	Diabetes *n* (%)	Diabetes with anemia *n* (%)	Diabetes without anemia *n* (%)	*p* value
Coronary angiography	56 (22.9)	25 (47.2)	31 (16.1)	<0.001
Percutaneous coronary intervention	34 (13.9)	15 (28.3)	19 (9.9)	0.001
Coronary artery bypass surgery	10 (4.1)	5 (9.4)	5 (2.6)	0.027
Arteriography	13 (5.3)	8 (15.1)	5 (2.6)	<0.001
Invasive treatment of chronic lower extremity ischemia^∗^	9 (3.7)	7 (13.2)	2 (1.0)	<0.001
Lower extremity amputations	2 (0.8)	1 (1.9)	1 (0.5)	0.328
Device therapies^∗∗^	9 (3.7)	3 (5.7)	6 (3.1)	0.385

^∗^Bypass surgery and endovascular treatment (percutaneous angioplasty with stenting, balloon angioplasty). ^∗∗^Implantable cardioverter defibrillator (ICD) and cardiac resynchronization therapy (CRT).

**Table 5:** Death risk factors in diabetic patients: the univariate and multivariate analysis.

Parameter/variables	HR	95% CI	*p* value
*Univariate analysis*			
Sex (female)	3.957	0.462–33.870	0.209
Age (years)			
70–79	1.985	0.124–31.848	0.628
≥80	10.771	1.202–96.531	0.034
Anemia	18.442	2.151–158.080	0.008
Heart failure	0.239	0.003–18.869	0.521
Coronary heart disease	1.803	0.330–9.853	0.496
Cancer	4.228	0.774–23.099	0.096
Acute coronary syndrome before age 60	2.619	0.306–22.449	0.380
Stroke before age 60	0	0	0.996
Atrial fibrillation	1.644	0.192–14.085	0.650
Chronic kidney disease	0	0	0.996
Pulmonary disease (asthma, COPD)	2.077	0.241–17.858	0.505
PAD	0.770	0.180–3.240	0.774
ESR	1.020	0.980–1.050	0.211

*Multivariate analysis*			
Anemia	15.232	1.767–131.295	0.013

ESR: erythrocyte sedimentation rate; CI: confidence interval; COPD: chronic obstructive pulmonary disease; HR: hazard ratio; PAD: peripheral artery disease.

## Data Availability

The reader can access the data by correspondence with the authors.
